# Acute Poisonings at a Regional Referral Hospital in Western Kenya

**DOI:** 10.3390/tropicalmed3030096

**Published:** 2018-09-03

**Authors:** Mitchel Otieno Okumu, Minal Naran Patel, Foram Rajnkant Bhogayata, Irene Awuor Olweny, Francis Okumu Ochola, Joshua Orungo Onono

**Affiliations:** 1 Department of Pharmacy, Jaramogi Oginga Odinga Teaching and Referral Hospital, Kisumu P.O. Box 849-40100, Kenya; minalmeghani@gmail.com (M.N.P.); forambhogayata10@gmail.com (F.R.B.); olwenyirene365@gmail.com (I.A.O.); 2 Department of Public Health, Pharmacology and Toxicology, Faculty of Veterinary Medicine, Nairobi P.O. Box 29053-00625, Kenya; joshua.orungo@uonbi.ac.ke; 3 Department of Pharmacology and Toxicology, School of Medicine, Moi University, Eldoret P.O. Box 3900-30100, Kenya; ochola.francis@yahoo.com

**Keywords:** acute poisoning, Western Kenya, snakebite, organophosphates

## Abstract

The emergency department (ED) of the Jaramogi Oginga Odinga Teaching and Referral Hospital (JOOTRH) handles many cases of poisoning. However, there is scant information on the factors, agents, and outcomes of poisoning at the hospital. The aim of this work was to determine the factors, agents, and outcomes of poisoning at JOOTRH. Records of patients who presented to JOOTRH with symptoms of poisoning between January 2011 and December 2016 were retrieved. Data on age, gender, offending agents, time, and season of exposure were collected. Information on the route of exposure, motive, and clinical symptoms of poisoning was also included. Other information included the laboratory evaluation, first aid measures, period of hospitalization, and outcome of poisoning. Mean, standard deviation, frequencies and bar graphs were used to describe the demographic factors of the study population. Multivariate logistic regression was used to determine the strength of association between risk factors and outcome of poisoning among patients. The level of significance for inferential analysis was set at 5%. There were 385 cases of poisoning: 57.9% (223/385) were male, 31.9% (123/385) were 13–24 years of age, and 83.9% (323/385) of exposures were in Kisumu County. The peak time of exposure was 6:00–00:00, and 23.6% (91/385) presented 1–4 h after exposure. About 62.9% (242/385) of the cases were due to accidental poisoning. Snakebites and organophosphates (OPPs) contributed to 33.0% (127/385) and 22.1% (85/385) of all cases, respectively. About 62.1% (239/385) of exposures were oral, and 63.9% (246/385) of all cases occurred in the rainy season. Additionally, 49.2% (60/122) of intentional poisoning was due to family disputes, and 16.1% (10/62) of pre-hospital first aid involved the use of tourniquets and herbal medicine. About 28.6% (110/385) of the victims were subjected to laboratory evaluation and 83.9% (323/385) were hospitalized for between 1–5 days. Other results indicated that 80.0% (308/385) responded well to therapy, while 7.3% (28/385) died, 68% (19/28) of whom were male. Furthermore, 39.3% (11/28) of the deaths were related to OPPs. Our findings suggest that the earlier the victims of poisoning get to the hospital, the more likely they are to survive after treatment is initiated. Similarly, victims of poisoning due to parental negligence are more likely to survive after treatment compared to other causes of poisoning, including family disputes, love affairs, snakebites, and psychiatric disorders. The management of JOOTRH should consider allocating resources to support the development of poison management and control.

## 1. Introduction

Poisoning is both a social and medical problem worldwide, and it is often a major cause of hospital admissions and fatalities [1]. According to estimates by the World Health Organization (WHO), there were about 200,000 poison-related fatalities in 2012, 80% of which occurred in low- and middle-income countries [2]. There is a large body of literature on poisoning in Kenya, covering aflatoxins [3], mushrooms [4], mercury [5], methanol [6], and pesticides [7], which have all been implicated as offending agents in human poisoning. Moreover, cestrum [8], cyanobacteria [9], lead [10], heavy metals [11], and Furadan (carbofuran) [12] intoxication have been reported, mostly in livestock and flamingos. Venomous snakebite injuries and factors associated with snake envenomation in some areas of Kenya have also been reported [13,14]. However, hospital-based data on acute poisoning in Kenya is scarce. Between 1980 and 2015, cases of poisoning have been reported in various hospitals in Kenya [15,16,17,18,19,20]. From these studies, organophosphates, organochlorines and other pesticides [15,16,17,20] have been reported as offending agents. Other offending agents reported include industrial and household products [18], kerosene [16,18,19], over-the-counter drugs [16] and rodenticides [20]. Children under 5 years of age and young adults have been identified as vulnerable groups [16,18,20] and a few studies have even estimated poison-related mortality to be between 3% and 45% of all presenting cases of poisoning [15,18,20]. It has also been reported that there is poor recognition and management of poisoning in some district hospitals in Kenya [17,20]. Having audited cases of poisoning at Kenyatta National Hospital (the main referral hospital in Kenya) over a period of one year, some workers postulated that their findings may mirror the state of acute poisoning in other parts of Kenya [18]. However, it may not be appropriate or accurate to generalize such findings given the complexities of different demographics and clinical settings. Jaramogi Oginga Odinga Teaching and Referral Hospital (JOOTRH) is a regional referral hospital in Western Kenya which handles many cases of poisoning. However, there is scant information on the factors, offending agents, and outcomes of acute poisoning at the facility. The aim of the present study was to determine the factors, offending agents, and outcomes of acute poisoning at JOOTRH over a 6-year period. The findings from this report are important for policy making with regard to allocation of resources and developing capacity for managing poisoning at the facility. Other health facilities with similar health systems may also benefit from the information.

## 2. Materials and Methods

### 2.1. Study Design

This was a retrospective record-based study of all cases of poisoning that were presented to the ED of JOOTRH from January 2011 to December 2016. Data was collected from charts of patients who were admitted at JOOTRH due to acute poisoning. A pre-structured proforma was used to extract data from treatment records of patients diagnosed with poisoning. Relevant files were reviewed for the year of poisoning, gender, age, place of residence, religion, marital and occupational status. The nature and motive of poisoning, the period of hospitalization, initial/final diagnosis, time lapse before treatment, and the types of poison, were also recorded. Data on the route of exposure, clinical presentation, laboratory findings, management, and outcome of poisoning, were also included.

### 2.2. Study Setting and Population

The study was conducted at JOOTRH in Kisumu County. The county is located 360 km north-west of Nairobi (−0.1022, 34.7617) and current census reports indicate that there are about 968,909 people residing in the county [21]. The facility is a regional referral hospital in Western Kenya [22]. Its catchment area extends to 10 counties in the Western Kenya region, namely Kisumu (population 968,909), Siaya (pop. 842,304), Homa Bay (pop. 749,331), Migori (pop. 256,086), Kisii (pop. 1,152,282), Kakamega (pop. 1,660,651), Vihiga (pop. 554,622), Bungoma (pop. 1,630,934), Busia (pop. 488,075), and Nandi (pop. 752,965) counties [21]. The hospital provides curative, preventive, promotive, and rehabilitative health services [22]. It has a bed capacity of 467, with a bed occupancy of about 94.8% [22]. 

### 2.3. Statistical Analysis

All the data was entered into an MS Excel spreadsheet and analyzed using the Statistical Package for the Social Sciences (SPSS) version 20.0 (IBM^®^, Armonk, NY, USA). Descriptive statistical measures, including mean, standard deviation, frequencies, and bar graphs, were used to describe the demographic factors of the study population. Multivariate logistic regression was used to determine the strength of association between risk factors and outcome of poisoning among patients. The level of significance for inferential analysis was set at 5%. 

### 2.4. Ethical Considerations

Ethical approval was obtained from the Ethical Review Committee (ERC) of JOOTRH (Ref: ERC.IB/VOL1/412, accreditation no: 01713) (Figure S1). Permission to conduct the study was obtained from the hospital administration. 

## 3. Results

### 3.1. Demographic Characteristics of Victims

There were 394 cases of poisoning retrieved from the hospital registry over the study period. Of these, 98% (385/394) had complete data to be evaluated. There was a total of 44,954 admissions at JOOTRH during the study period, with poisoning accounting for at least 0.9% of all cases. Most cases occurred in 2012, and accounted for 22.9% (88/385) of all poisonings, while the least number occurred in 2011, accounting for 9.1% (35) of all cases. There were 70, 59, 68, and 65 cases of poisoning in 2013, 2014, 2015, and 2016, respectively. The mean age of poisoned patients was 22.60 ± 3.41 years (males: 24.14 ± 16.15, females: 20.48 ± 14.62 (overall range: 10 months to 84 years). Other demographic characteristics of the victims are summarized in Table 1. 

**Figure 1:**
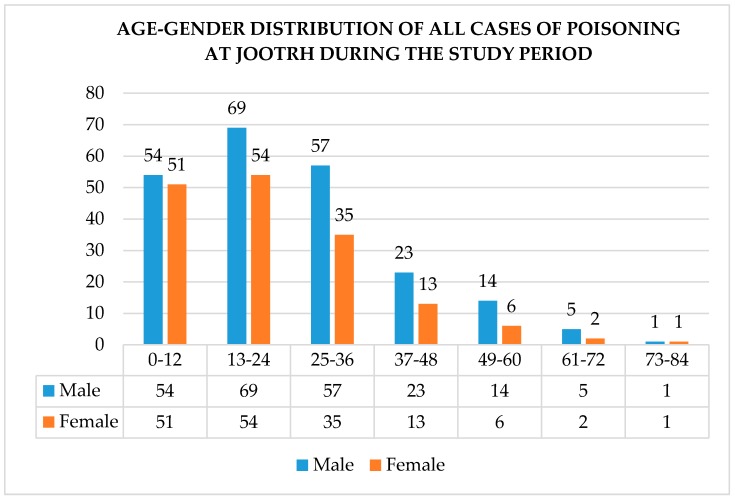
Age–gender distribution of cases of poisoning at JOOTRH during the study period.

Figure 1 is a summary of the age–gender distribution of victims of poisoning at JOOTRH during the study period.

### 3.2. Location and Time of Exposure, Referring Facilities, and the Time Taken to Get to the Hospital 

The location and time of exposure, referring facilities, and time taken by victims to get to the hospital, are summarized in Table 2.

### 3.3. Offending Agents, Routes of Exposure, and Season of Poisoning

The offending agents, routes of exposure and season of poisoning among victims at JOOTRH during the study period are summarized in Table 3.

Most cases of poisoning occurred during the month of May (42/385 (10.9%)) while January registered the least number of cases (18/385 (4.7%)) (data not shown).

**Figure 2:**
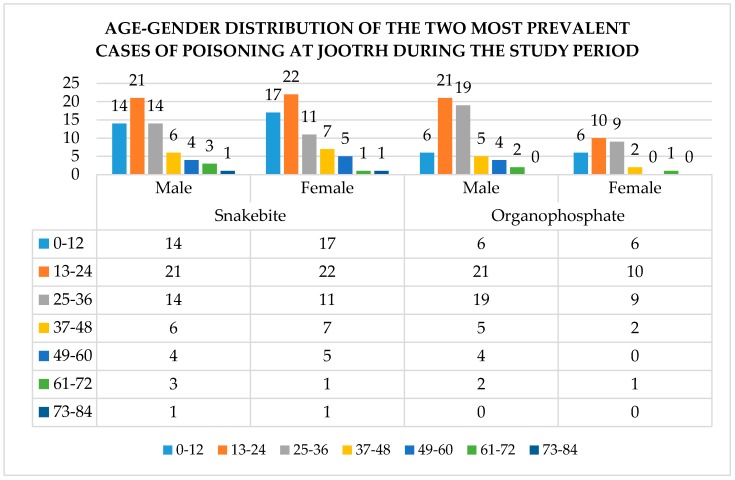
Distribution of the two most common causes of poisoning in the study area.

The age–gender distribution of the two major poisons among victims presenting to JOOTRH, during the study period, is summarized in Figure 2.

### 3.4. Circumstance and Reasons for Poisoning 

The circumstances and reasons for poisoning among victims presenting to JOOTRH during the study period are summarized in Table 4.

### 3.5. Pre-Hospital First Aid among Victims and Identification of Offending Agents by Emergency Care Personnel

Pre-hospital measures initiated by victims of poisoning and methods used by emergency care personnel to identify offending agents are summarized in Table 5.

### 3.6. Admission Status and Outcome of Treatment of Victims at the Intensive Care Unit (ICU) 

Thirty-three victims, accounting for 8.6% of all cases, were admitted to the intensive care unit (ICU) for further management. Among the ICU cases, organophosphates and amitraz were the main offending agents, accounting for 51.5% (17/33) and 18.2% (6/33) of all cases, respectively. Cyanide (from cassava ingestion), drugs (indomethacin and aspirin), and unknown poisons were responsible for two ICU admissions each (2/33 (6.1%)). Carbon monoxide, snakebite, ethanol, and herbal medicine were responsible for one admission each. Moreover, 27.3% (9/33) of all ICU admissions died. Organophosphates and amitraz accounted for 44.4% (4/9) and 22.2% (2/9) of all ICU deaths, respectively, while snakebite, cyanide (from cassava ingestion), and herbal medicine intoxication accounted for one ICU death each (11.1%).

### 3.7. Length of Hospital Stay, Status of Treatment, Mortality Causing Offending Agents, and Demographic Features of Victims Who Succumbed to Poisoning

The mean length of hospital stay among victims was 3.7 ± 3.9 days. Around 83.9% (323/385) of all victims spent 1–5 days in the hospital, while 10.6% (41/385) spent 6–10 days. Another 3.1% (12/385) of victims spent 11–15 days, while 0.5% (2/385) spent between 16 and 20 days. Additionally, 1.8% (7/385) of all victims spent >20 days in the hospital.

Following treatment, 80% (308/385) of all victims improved, 7.3% (28/385) died, and 68% (19/28) of all deaths were male. Furthermore, 25% (7/28) of all fatalities were 0–12 years of age, and another 25.0% (7/28) were between 13 to 24 years of age. 

Overall, organophosphates accounted for 39.3% (11/28) of all fatal outcomes. Others were herbal medicine 14.3% (4/28), snake bites 14.3% (4/28), food poisoning 7.1% (2/28), and amitraz 7.1% (2/28). Corrosives, ethanol, insect repellent, kerosene, and an unknown poison accounted for 1 victim each (3.6%). The symptoms of poisoning observed among victims and laboratory evaluations that were carried out are summarized in Table 6.

### 3.8. Treatment of the Two Major Poisons and Effect on the Outcome

#### 3.8.1. Snakebite

**Figure 3:**
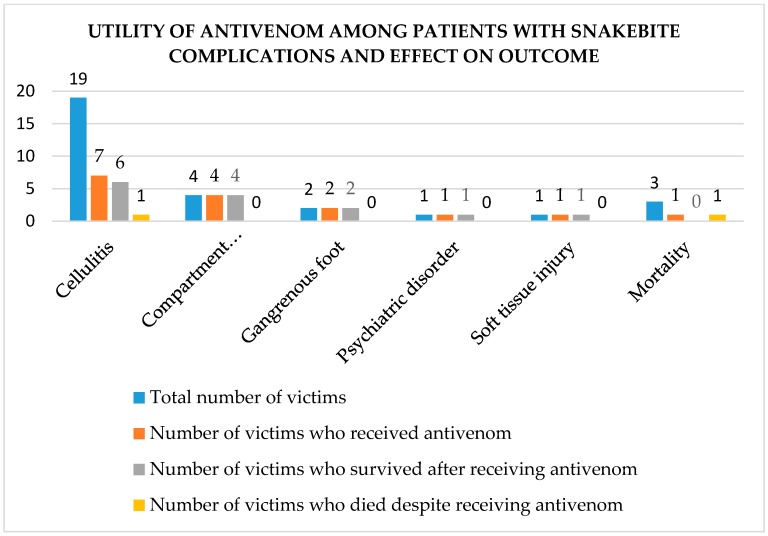
Utility of antivenom and outcome among patients with snake bite related complications.

Figure 3 is a summary of the utility of antivenom in cases of snakebite related complications among victims who presented to JOOTRH during the study period.

Of the remaining 97 cases of snakebite (those that had no complications), antivenom was used in 57 cases (58.8%). Most victims described the offending snakes on the basis of their color. From the description provided by victims, black snakes were implicated in 37.8% (48/127) of all snakebite cases, brown snakes in 11.0% (14/127) of all cases, and green snakes in 12/127 (9.4%) of all cases. Moreover, grey snakes were implicated in 0.02% (2/127) of all cases, and a snake described as white and brown with black spots was implicated in 2/127 (0.02%) of the cases. Victims were not able to identify the snakes (even based on color) in 38.6% (49/127) of all cases of snakebites. There was only one case of snakebite where a dead snake specimen was presented to the emergency department of JOOTRH, and was identified as a puffadder (*Bitis arietans*) (‘foo’ in the local Luo language). 

#### 3.8.2. Organophosphates

**Figure 4:**
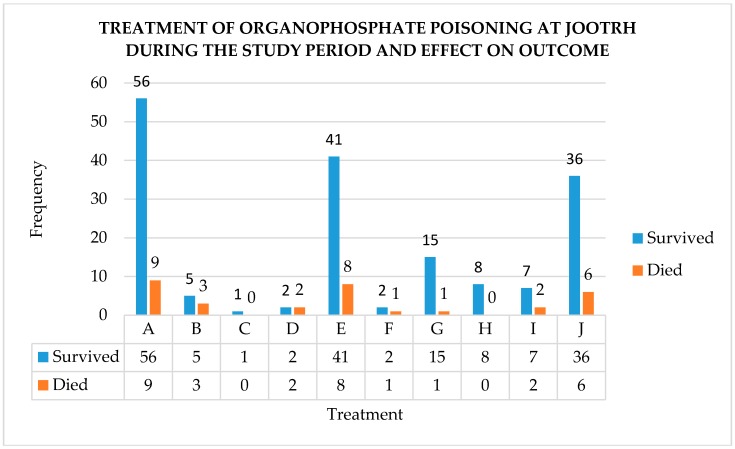
Frequency of use and outcome of different methods of managing organophosphate poisoning at JOOTRH, where A: supportive care (IV fluids), B: supportive care (oxygen via mask), C: supportive care (oxygen via nasal catheter), D: supportive care (methodology not specified), E: atropine, F: pralidoxime, G: atropine and pralidoxime, H: forced diuresis, I: activated charcoal, J: gastric lavage.

Figure 4 is a summary of the frequency of use and the outcome of different methods of managing organophosphate poisoning. 

### 3.9. Associations between Exposure Factors and Outcome of Poisoning Amongst Patients

Results of the analysis of the association between exposure factors and outcome of poisoning amongst patients is summarized in Table S3. The factors that were significantly associated with survival amongst victims of poisoning at JOOTRH were related to reasons for poisoning and the time taken by patients before seeking medical assistance. Factors associated with the nature of poisoning had no significant effect on the outcome of exposure to poisonous agents. From this analysis, those patients who sought medical assistance within the first 6 h and between 6 and 12 h after exposure to poisonous agents were more likely to survive after treatment as compared to those who presented more than 12 h after exposure to poisons. Similarly, those patients who got poisoned due to parental negligence were more likely to survive after treatment as compared to other causes of poisoning including family disputes, love affairs, snakebites, and psychiatric disorders.

## 4. Discussion

The society in Western Kenya lays more socioeconomic burden on males than it does on females. However, unemployment and poverty in the region occasioned by dwindling resources mean that it is becoming difficult for men to meet societal obligations. This may predispose them to self-harm by poisoning, and may partly explain why most victims in the study area were male.

Our observation that teenagers and young adults were the most vulnerable group disagrees with the findings of previous authors [18,20]. Therefore, it may be suggested that the age of exposure to toxic substances in Kenya may dependent on the geographical location of the victims.

The reasons for the relatively high number of cases of poisoning from Kisumu East and Nyando sub-counties may have something to do with the socioeconomic activities in these areas. These areas are renowned for rice, maize, and sugarcane farming. Livestock farming is also practiced. On the one hand, victims of snakebites may have been exposed in the course of their day-to-day activities in farms. On the other hand, toxic exposure to organophosphates may be due to the widespread use of agrochemicals in the region. Poisoning with agrochemicals has generally been reported in populations that are predominantly agrarian [7].

The range of pre-hospital measures adopted by victims may imply an undocumented pattern of health-seeking behavior among the population. Most of these appear to be enshrined in customary practice and folklore. Notwithstanding, there is an expanding body of medical knowledge that continues to cast aspersions on the efficacy of some of these first aid measures. Incisions, suction, heat, ice, alcohol, electric shocks, and topical chemicals/herbals are all contraindicated in snakebites [23]. In our study, there was a bizarre case of a 31-year-old female from Kamrongo (Kisumu East), who was bitten by a snake on her left leg. In addition to having a tourniquet tied and incisions made at the site of the bite, she also resorted to swallowing a raw egg. It is unclear how these measures were meant to help prevent the spread of venom. What is even more damning is that despite presenting with pitting edema of the lower left leg, she requested discharge against medical advice after spending just two days in the hospital. There was no way of tracking her progress post-discharge, as no mechanism of patient follow-up exists at the facility.

Appropriate pre-hospital treatment of snakebite includes removal of constrictive items, pressure immobilization, basic life support, pain control, and hydration [23]. Emesis is also contraindicated in the event of paraffin/petroleum distillate poisoning. Nonetheless, victims were not aware of this, and often used raw eggs or porridge to induce vomiting after exposure to these agents. The result was aspiration pneumonia, which further complicated the management of the cases.

The length of stay of a majority of the victims was consistent with the findings of a study in India [24]. However, it was interesting to note that some of the victims may have been discharged before fully recovering. One striking case was that of a 29-year-old female from Masogo (Nyando sub-county) who declined fasciotomy, despite having developed compartment syndrome secondary to a snakebite. Her decision to decline treatment was anchored on the fact that she had nobody to take care of her two young children. In her opinion, a prolonged stay in the hospital following the procedure (fasciotomy) would jeopardize the wellbeing of her children.

Animal epidemiological surveys [25] have reported that some of the venomous snakes in the region include black mamba (*Dendroaspis polylepis*), eastern Jameson’s mamba (*Dendroaspis jamesoni*), puffadder (*Bitis arietans*), and black-necked spitting cobra (*Naja nigricollis*). Others include the boomslang *(Dispholidus typus)*, mole viper *(Actractaspis microlepidota),* the large brown spitting cobra (*Naja ashei*), forest cobra *(Naja melanoleuca)*, gaboon viper *(Bitis gabonica)*, rhino viper *(Bitis nasicornis)*, Egyptian cobra (*Naja haje*), and the night adder *(Causus rhombeatus)*. Some of these snakes may have been responsible for the hospital admissions in our study. Moreover, the large number of complications arising from snakebite may suggest that emergency care personnel may not be adequately equipped and trained to manage snakebites.

Combining atropine and pralidoxime in the management of organophosphate poisoning is controversial. On one hand, skeptics argue that there is no benefit in combining the two [26]. On the other hand, proponents argue that the use of pralidoxime lowers the dose requirements of atropine, reduces the requirement of intubation and ventilator support, decreases the development of muscle weakness, and minimizes the occurrence of organophosphate-associated pneumonia [27]. In our case, the high survival rate associated with combining atropine and pralidoxime as compared to using atropine alone seems to corroborate the latter argument. Notwithstanding, the availability of mechanical ventilation and critical care in our setting was wanting. This was exemplified by the case of a 65-year-old male patient exposed to organophosphate poisoning, who died after he could not secure one of four active ICU beds.

It has been reported elsewhere [20] that emergency-care personnel tend to make a default diagnosis of organophosphate poisoning. This holds true particularly when they are not sure of the offending agent. The same was replicated in our setting (albeit to a lesser degree). On further analysis, we found that most of these instances involved amitraz intoxication. Miosis, respiratory depression, and bradycardia are hallmarks of both amitraz and organophosphate intoxication. Thus, it is easy to see why this was a common cause of misdiagnosis. Nonetheless, this phenomenon often led to inappropriate use of important antidotes, such as atropine and pralidoxime. These observations are a clarion call for poison management protocols at the facility.

This was a single center study, and thus, it may not be possible to generalize our findings to other demographics and clinical settings. The mortality rate we have reported may most likely be an underestimate of the true burden of acute poisoning in the Western Kenya region. This is because our study was not able to include victims who had sought treatment in neighboring health facilities and were never referred to JOOTRH. It is also possible that severe cases may have succumbed to effects of the poison at home without presenting to our facility. There was no mechanism in place to facilitate patient follow up. This made it impossible to ascertain the actual outcomes of discharged patients. Moreover, victims of poisoning, such as those exposed to snakebites, may have sought treatment outside the formal healthcare system. Thus, the observed number of cases may be an underestimate of the true incidence of snakebites in the Western Kenya region.

We had to reject some files that were devoid of relevant clinical and sociodemographic information. Rejection of files was not frequent, and the effect may have been minimal. In spite of these shortcomings, our work provides useful information on the factors, offending agents, and outcomes of acute poisoning at JOOTRH, a major referral hospital in Western Kenya. This information may be useful in shaping policy on the need for allocation of resources and development of poison management capacity at JOOTRH.

## 5. Conclusions

Our findings suggest that the earlier the victims of poisoning get to the hospital, the more likely they are to survive after treatment is initiated. Similarly, victims of poisoning due to parental negligence are more likely to survive after treatment compared to other causes of poisoning including family disputes, love affairs, snakebites and psychiatric disorders. The management of JOOTRH should consider allocating resources to support development of poison management and control.

## Figures and Tables

**Table 1:** Demographic characteristics of victims of poisoning at Jaramogi Oginga Odinga Teaching and Referral Hospital (JOOTRH) during the study period.

Demographic Factor	*n* = 385
**Gender**	
Male	223 (57.9%)
Female	162 (42.1%)
Age	
0–12	105 (27.3%)
13–24	123 (31.9%)
25–36	92 (23.9%)
37–48	36 (9.4%)
49–60	20 (5.2%)
61–72	7 (1.8%)
73–84	2 (0.5%)
**Marital status**	
Married	139 (36.1%)
Child	117 (30.4%)
Single	90 (23.4%)
Unknown	29 (7.5%)
Divorcees, widows, widowers	10 (2.6%)
**Occupation**	
Self-employed	107 (27.8%)
Students	89 (23.1%)
Child	77 (20.0%)
Unemployed	39 (10.1%)
Formally employed	33 (8.6%)
Casual laborers	11 (2.9%)
Unknown	29 (7.5%)

**Table 2:** Location of exposure, referring facilities, exposure time, and time to hospital.

Variable	Frequency (*n* = 385)
County	
Kisumu	323 (83.9%)
Siaya	28 (7.3%)
Vihiga	11 (2.9%)
Homabay	8 (2.1%)
Nandi	7 (1.8%)
Other ^a^	8 (2.1%)
Sub-county *	
Kisumu East	190 (58.8%)
Nyando	43 (13.3%)
Kisumu West	39 (12.1%)
Nyakach	18 (5.6%)
Kisumu Central	15 (4.6%)
Seme	11 (3.4%)
Muhoroni	7 (2.2%)
**Referring facilities**	
Public hospitals	74 (81.3%)
Private hospitals	11 (12.1%)
Mission hospitals	6 (6.6%)
**Time of exposure**	
0600–1159	49 (12.7%)
1200–1759	60 (15.6%)
1800–2359	91 (23.6%)
0000–0559	20 (5.2%)
No data	165 (42.9%)
**Time to hospital**	
<1 h	10 (2.6%)
1–4 h	91 (23.6%)
4–8 h	49 (12.7%)
8–12 h	19 (4.9%)
12–16 h	8 (2.1%)
16–20 h	8 (2.1%)
20–24 h	19 (4.9%)
>24 h	5 (1.3%)
Unknown	176 (45.7%)

**^a^** Includes Kakamega, Migori, Bungoma, Busia, and Kisii counties, * Sub-counties within Kisumu County.

**Table 3:** Offending agents, routes of exposure and season of poisoning.

Variable	Frequency (*n* = 385)
**Offending agents**	
Snake poison	127 (33%)
Organophosphates ^a^	85 (22.1%)
Kerosene/paraffin	29 (7.5%)
OTC ^b^ and prescription drugs ^c^	19 (4.9%)
Amitraz	18 (4.7%)
Food	16 (4.2%)
Ethanol	15 (3.9%)
Corrosive chemicals	15 (3.9%)
Herbal medicine	9 (2.3%)
Rodenticides	8 (2.1%)
Insect repellant	8 (2.1%)
Others ^d^	19 (4.9%)
**Route of exposure**	
Oral/ingestion	239 (62.1%)
Inoculation	131 (34.0%)
Mucocutaneous	9 (2.3%)
Inhalation	5 (1.3%)
Unknown	1 (0.3%)
**Season of poisoning**	
Long rainy season	172 (44.7%)
Short rainy season	74 (19.2%)
Cool dry season	78 (20.3%)
Hot dry season	61 (15.8%)

^a^ Includes diazinon, ‘Gladiator’, ‘Zylon’, unidentified acaricides, unidentified tick poison, and unidentified bed-bug poison. ^b^ Over the counter (OTC) medication; includes paracetamol, unidentified cough tablets, aspirin, and loperamide. ^c^ Prescription drugs; includes carbamazepine, indomethacin, antiretrovirals (zidovudine/lamivudine, nevirapine; AZT/3TC/NVP, tenofovir/lamivudine/efavirenz; TDF/3TC/EFV), and co-trimoxazole tablets (CTX). Also includes metronidazole and ciprofloxacin tablets, unidentified antihypertensives, and unidentified proton pump inhibitors. ^d^ Includes poisonous berries, petroleum distillates (turpentine and diesel), and household products. Also includes carbon monoxide, poisonous cassava, and mushrooms, potassium permanganate, street glue, thymol, and deltamethrin.

**Table 4:** Circumstances and reasons for poisoning among victims at JOOTRH.

Variable	Frequency (*n* = 385)
**Circumstance**	
Accidental	242 (62.9%)
Suicidal	122 (31.7%)
Homicidal	17 (4.4%)
Suicidal and homicidal	1 (0.3%)
Unknown	3 (0.8%)
**Reasons for poisoning**	
Accidentally bitten by snakes	127 (33.0%)
Domestic quarrels	68 (17.7%)
Parental negligence	44 (11.4%)
Poor food handling/preparation	17 (4.4%)
Alcohol misuse	14 (3.6%)
Psychiatric disorders	12 (3.1%)
Love affairs	11 (2.9%)
Curiosity	7 (1.8%)
Traditional beliefs	5 (1.3%)
Unemployment	4 (1.0%)
Unknown	51 (13.2%)
Others ^a^	25 (6.5%)

^a^ Includes college dismissal, exam failure, financial problems, HIV status, poorly-labelled medicine, occupational hazard, self-treatment, and unplanned pregnancy.

**Table 5:** Pre-hospital first aid measures among victims and methods used to identify offending agents.

Variable	Frequency (*n* = 385)
**First aid measure**	
Tying a tourniquet	10 (2.6%)
Use of herbal medicine	10 (2.6%)
Use of milk	7 (1.8%)
Use of raw eggs	7 (1.8%)
Use of both raw eggs and milk	4 (1.0%)
Incisions	2 (0.5%)
Torniquet, incisions, and herbal medicine	2 (0.5%)
Incisions and herbal medicine	2 (0.5%)
None	323 (84%)
Others ^a^	18 (4.7%)
**Mode of identifying offending agents**	
Container	104 (27.0%)
The color of the offending animal	83 (21.6%)
Odor only	14 (3.6%)
Container and odor	8 (2.1%)
History from informant	11 (2.9%)
Identification of the food source	12 (3.1%)
Fang marks	12 (3.1%)
None	134 (34.8%)
Others ^b^	7 (1.8%)

^a^ Includes tying a tourniquet and limb elevation; tying a tourniquet and impregnating a cloth with charcoal; tying a tourniquet and making incisions; use of porridge; cleaning the wound; use of herbal medicine and washing with water; rinsing the mouth; applying Vaseline. ^b^ Includes a description of the animal; dead animal brought to the hospital; fang marks; pruritus; and the hissing sound of a snake.

**Table 6:** Symptomatology and laboratory evaluations among victims.

Variable	Frequency
**Clinical presentation (*n* = 385)**	
Neurological ^a^ and gastrointestinal ^b^	80 (20.8%)
Neurological ^a^ only	71 (18.4%)
Neurological ^a^ and swelling	53 (13.8%)
Gastrointestinal ^b^ only	53 (13.8%)
Neurological ^a^, gastrointestinal ^b^, and cardiopulmonary ^c^	28 (7.3%)
Neurological ^a^ and cardiopulmonary ^c^	26 (6.8%)
Swelling only	14 (3.6%)
Neurological ^a^ and bleeding	13 (3.4%)
Neurological ^a^, swelling, and bleeding	12 (3.1%)
Asymptomatic	4 (1.0%)
Others	31 (13.8%)
**Laboratory evaluation (*n* = 110)**	
RBS only	28 (25.5%)
FHG, serum electrolytes, and BUN	17 (15.5%)
FHG, serum electrolytes, and Scr	9 (8.2%)
FHG, serum electrolytes, and LFTs	7 (6.4%)
FHG only	7 (6.4%)
Abdominal ultrasound	4 (3.6%)
FHG and serum electrolytes	4 (3.6%)
Others ^x^	34 (30.9%)

^a^ Includes convulsions, headache, impaired consciousness, slurred speech, radiating pain, paresthesia, burning sensation at the site of the bite, cough, sweating, malaise, dizziness, hyperthermia, ‘pins and needles’ sensation, blurred vision, and loss of hearing. ^b^ Includes abdominal pain, nausea, and vomiting. ^c^ Includes dyspnea. RBS: random blood sugar, FHG: full hemogram, BUN: blood urea nitrogen, Scr: serum creatinine, LFTs: liver function tests. ^x^ Includes chest X-ray, skull X-ray, cerebrospinal fluid tap, electroencephalography, stool examination, electrocardiography, urinalysis, and blood film for malaria.

## Supplementary Materials

The following are available online at http://www.mdpi.com/2414-6366/3/3/96/s1, Figure S1: Ethical Approval Form; Table S2: Reasons for deliberate poisoning and distribution of victims based on gender and age; Table S3: Multivariate analysis of poisoning exposure factors and the survival of victims who presented to JOOTRH during the study period.
