# Supplementary material for: Acute Poisonings at a Regional Referral Hospital in Western Kenya
Source: Trop Med Infect Dis. 2018 Sep 3;3(3):96. doi: 10.3390/tropicalmed3030096 (PMC6161120; doi:10.3390/tropicalmed3030096)
Supplement: Supplementary file 1 [file tropicalmed-03-00096-s001.pdf]

Supplementary information

Telegrams: "MEDICAL", Kisumu
Telephone: 057-2020801/2020803/2020321
Fax: 057-2024337
E-mail: ercjootrh@gmail.com
When replying please quote

JARAMOGI OGINGA ODINGA TEACHING & REFERRAL HOSPITAL
P.O. BOX 849
KISUMU

Ref: ERC.IB/VOL.1/412

Date: 28th February, 2018

Dr. Mitchel Otieno Okumu
KISUMU

Dear Mitchel,

RE: **FORMAL APPROVAL TO CONDUCT RESEARCH ENTITILED:-** ***ACUTE POISONINGS AT A REGIONAL REFERRAL HOSPITAL IN WESTERN KENYA***

The JOOTRH ERC reviewed your protocol and found it ethically satisfactory. You are therefore permitted to commence your study immediately. Note that this approval is granted for a period of one year (28th February, 2018 to 28th February, 2019). If it is necessary to proceed with this research beyond approved period, you will be required to apply for further extension to the committee.

Also note that you will be required to notify the committee of any protocol amendment(s), serious or unexpected outcomes related to the conduct of the study or termination for any reason.

In case the study site is JOOTRH, kindly report to the Chief Executive Officer before commencement of data collection.

Finally, note that you will also be required to share the findings of the study in both hard and soft copies upon completion.

The JOOTRH – ERC takes this opportunity to thank you for choosing the Institution and wishes you the best in your endeavours.

Yours sincerely,

JOOTRH ETHICS & REVIEW COMMITTEE
P. O. Box 849 - 40100
KISUMU

WILBRODA N. MAKUNDA
SECRETARY- ERC
JOOTRH - KISUMU

Figure S1. Ethical Approval Form.

Table S2. Reasons for deliberate poisoning and distribution of victims based on gender and age.

Variable	Frequency (n = 122)
**Reasons**	
Family/domestic dispute	60 (49.2%)
Love affairs	10 (8.2%)
Psychiatric disorders	9 (7.4%)
Unemployment	3 (2.5%)
Police cases/ harassment	2 (1.6%)
Unplanned pregnancy	2 (1.6%)
Financial problems	2 (1.6%)
College dismissal	2 (1.6%)
Death of wife	1 (0.8%)
Work related issues	1 (0.8%)
Unknown	30 (24.6%)
**Gender**	
Male	82 (67.2%)
Female	40 (32.8%)
**Age**	
0-12	4 (3.3%)
13-24	53 (43.4%)
25-36	44 (36.1%)
37-48	12 (9.8%)
49-60	7 (5.7%)
61-72	2 (1.6%)

Table S3. Multivariate analysis of poisoning exposure factors and the survival of victims who presented to JOOTRH during the study period.

Description of factors and reasons for poisoning	Estimate	Std error	Wald Chi-Square	Sig
**Nature of poisoning**				
a) Suicidal	0.076	0.200	0.146	0.702
b) Accidental	-0.241	0.295	0.666	0.415
c) Homicidal	0[a]	.	.	.
**Possible reasons for poisoning**				
a) Family dispute	0.318	0.275	1.344	0.246
b) Parental negligence	0.724	0.360	4.060	0.044
c) Snake bites	0.559	0.300	3.472	0.062
d) Psychiatric disorders	0.662	0.354	3.501	0.061
e) Love affairs	0[a]	.	.	.
**Referring facility**				
a) Within Kisumu County	-0.130	0.092	1.988	0.159
b) Outside Kisumu County	0[a]	.	.	.
**Hours before seeking medical assistance**				
a) < 6 h	-0.225	0.092	5.949	0.015
b) Between 6 and 12 h	-0.269	0.118	5.226	0.022
c) >12 h	0[a]	.	.	.
